# Full-Arch Guided Restoration and Bone Regeneration: A Complete Digital Workflow Case Report

**DOI:** 10.3390/healthcare11091301

**Published:** 2023-05-02

**Authors:** Claudia Todaro, Michael Cerri, Ruggero Rodriguez y Baena, Saturnino Marco Lupi

**Affiliations:** 1 School of Dentistry, Department of Clinical Surgical, Diagnostic and Pediatric Sciences, University of Pavia, 27100 Pavia, Italy; 2 Private Practice, 29011 Borgonovo Val Tidone, Italy

**Keywords:** digital workflow, 3D printing, 3D surgical template, digital smile design, VOD, PRF, GBR, prosthodontics, digital technology, computer-aided design

## Abstract

Objective: complex rehabilitations present multiple difficulties, regarding both the planification of the surgery and the design of the prothesis. A digital approach can support the workflow, as well as the degree of intraoperative precision, and improve the long-term prognosis. Methods: A surgical guide was designed for implant placement. An extensive regeneration of the upper jaw was performed with contextual implant insertion, and a delayed load rehabilitation was chosen. After four months, a second surgery and a simultaneous soft tissue augmentation was performed, and a 3D-printed temporary restoration was placed. After another two months, new dental and facial scans, smile design, and facial bite registrations were obtained. Upper and lower dentures were built using an exclusively digital workflow. Both metal substructures were passivated and cemented in one session; in the following appointment, the aesthetic and occlusal checks were carried out. During the third visit, both prostheses were delivered. Results: Careful case planning and the surgical guide made it possible to achieve primary stability and acceptable emergence profiles in an extremely reabsorbed upper jaw. Leukocyte-Platelet Rich Fibrin (L-PRF) made the extensive bone regeneration more approachable and lowered the post-operative pain and swelling, while speeding up the soft tissue healing process. During the re-entry surgery, the volumes of soft tissues were increased to improve aesthetics, and the amount of keratinized gingiva around the six implants was also increased. Smile design and facial scans have provided the means to create acceptable aesthetics and function in a few sessions with minimal patient discomfort. Conclusions: Computer-assisted implantology is a safe and precise method of performing dental implant surgery. Preliminary studies have a high degree of accuracy, but further studies are needed to arrive at a fully digital clinical protocol at all stages.

## 1. Introduction

Nowadays, there is a lot of scientific evidence that confirms that the implant-supported fixed prosthesis is a favorable choice in edentulous patients, or in patients with terminal dentition, in relation to the high success rate in the medium-long term [1,2]. Recent studies in the literature [3,4] have evaluated the survival and complication rates of fixed dental prostheses on implants showing high 5-year survival rates, ranging from 97.1% to 99%.

These valuable data are deduced from the analysis of samples with long-term follow-up resulting from preoperative case studies, which made it possible to select the ideal type of prosthesis, implant components, and implant material that avoids incurring premature failures that occur due to technical or mechanical complications.

The most frequent complications include fractures of the abutments, of the prosthetic screw or, more rarely, of the fixture itself. A scrupulous diagnosis that considers the greatest number of anatomical and prosthetic-limiting variables is necessary to improve the prognosis and the predictability of the treatment. To this end, the pre-visualization is used today through set-ups and wax-ups, in order to understand what the best biomechanical relationships may be, and to identify the ideal three-dimensional position of the implant fixtures, orienting them as best as possible according to the emergence profile of the prosthesis and of the specific functional determinants of the case.

The advent of 3D radiological instruments with low radiation dose emission, such as CBCT [5,6], allow to study the anatomical structures in detail and perform, by means of dedicated software, surgical-prosthetic planning of complex cases [7].

Cone beam computed tomography (CBCT) allows to analyze volumetric data of jaw bones, dental elements, and noble anatomical structures, with limited radiation doses. The processing of these DICOM files allows, in addition to the radiological diagnosis, the 3D planning of various intraoperative procedures, including surgical and implant procedures. The transfer of these plans to the operating field can be achieved through assisted navigation systems, or through printed guidance devices, with the use of biocompatible materials and 3D printers [6,8,9,10].

Other key data for planning is obtained from the intraoral scan. This represents a reliable method [11] which allows to reduce handling errors [12], as well as cumulative ones, reducing the sensitivity of the procedure related to the operator’s manual dexterity. If properly managed, it is more appreciated by patients, as it is more comfortable than the traditional impression and reduces chair time [13]. The accuracy of the intraoral scan, compared to the analog impression of the dental arches, has recently been evaluated in several studies. From the prosthetic point of view, the accuracy of the detection at the level of the abutment-implant connection was evaluated, demonstrating that digital impressions for full-arch implant-supported prostheses are as accurate as conventional impressions [14].

Other studies have highlighted a greater accuracy in favor of intraoral scanners in the case of high angulation degrees of the fixtures. The greatest number of interferences with the digital method is linked to the thickness of the soft tissues; in other words, the accuracy decreases the more the implant is deepened into the soft tissues [15,16,17,18].

The possibility of routinely operating through guided surgery techniques through 3D printing stereolithographic surgical guides is increasingly concrete [19]. This procedure allows the clinician to precisely plan the implant’s placement, taking into account the needs of the future prothesis [20,21,22]. The degree of reliability and precision of this method has been validated by the studies conducted so far.

Some works have stated that the template-guided implantation offers a high degree of accuracy, even in the presence of different configurations of the residual dentition or the edentulous arch. Recent preliminary studies [23] show a degree of accuracy of the templates in the presence of total edentulism, comparable to that achievable with intercalated edentulism. Similar data are due to the management of the exclusively mucosal support through digitally preconfigured anchor pins.

The clinical benefit of this type of approach is expressed at a surgical level, but it finds great advantage, above all, at a prosthetic level [24,25]. Applications driven by digital design and 3D printing have found primary application in orthopedics. Dentistry has benefited from the first studies in the orthopedic field and is positioning itself as a branch of medicine that, first of all, is making its workflow digital in daily practice, in the public and private sectors, providing a greater level of accuracy and reproducibility, even at the microscopic level [19,26].

The clinical approach, by means of a fully digital workflow, is a very topical and debated research area. Further studies are still needed to understand, for example, whether one technique is better than another at the production level, e.g., milling v/s 3D printing [27].

However, there is no doubt that the set and connection of the multiple technologies available today has diversified the process for reaching the diagnosis and the implant-prosthetic treatment plan, radically changing the way in which the workflow and communication with the team and the patient are managed, as well as the ability to plan and execute complex cases in a more accurate and predictable way. These tools offer a high diagnostic and therapeutic potential and the ability to preview prosthetically guided surgical options, reducing prosthesis timing, with the opportunity to deliver the provisional prosthesis at the end of the surgical session [28]. Despite the innumerable advantages, it is, however, clear that this approach requires in-depth digital knowledge and studies that allow for the management of complex cases to be dealt with in a more protocolled way.

The clinical case presented below offers points for scientific reflection, as it illustrates a completely digital approach to a complex screw-retained implant-prosthetic rehabilitation. This case of severe atrophy has been approached using the most recent tools that the digital world can offer. Starting from the digital planning of the case, passing through the creation of the 3D-printed surgical guide to the analysis of the prosthetic parameters, such as the OVD (Occlusal Vertical Dimension), phonetics etc. have been obtained using exclusive digital tools [29].

## 2. Clinical Case

**Figure 1:**
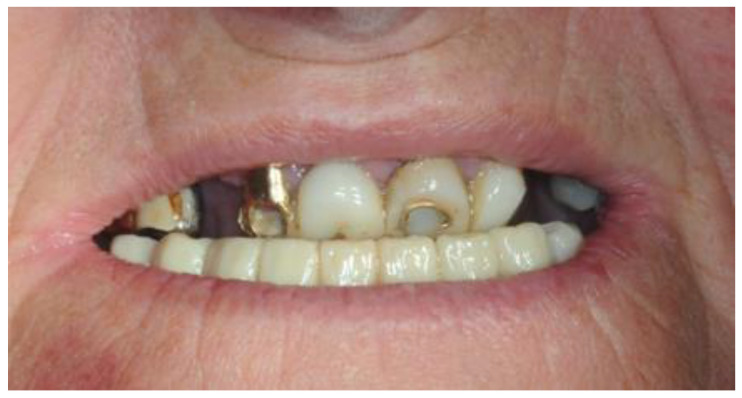
Pre-operatory frontal picture.

The 74-year-old patient came to our attention for a rehabilitation treatment in the presence of arches in the terminal dentition phase. The history indicated good general health, and the patient was classified as ASA II (classification of the anesthesiologic risk by the American Society of Anesthesiologists), due to mild hypertension. Extraoral physical examination showed good facial symmetry, with a discrepancy of less than 4 mm between the maxillary and mandibular midline. The patient with underrepresented soft tissues and thin lips did not have adequate smile arch (Figure 1).

**Figure 2:**
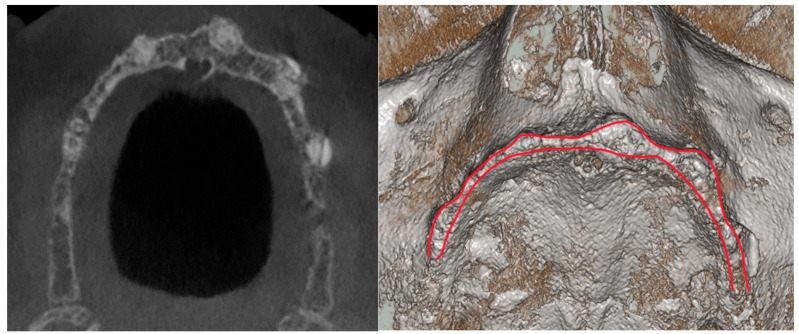
Pre-operatory CBCT. Detail of the reabsorbed maxilla.

The original treatment plan for the patient was to remove all residual elements in the mandible and maxilla, including an included 3.4, then rehabilitate the upper arch with a full removable denture, and the lower arch with a fixed immediate loaded prosthesis on four implants. This choice was agreed upon mainly to maintain lower costs. During the treatment phase, and following the delivery of the lower fixed provisional prosthesis, the patient reconsidered the option of a fixed prosthesis on implants also in the upper arch. The three-dimensional Cone-Beam Computer-Tomography (CBCT) examination highlighted a serious picture of maxillary atrophy (Figure 2) in the presence of hyper-pneumatization of the maxillary sinus. In the absence of signs and symptoms of sinusitis, it was decided, in accordance with the will and informed consent of the patient, to undertake a full-arch rehabilitation program, planning bone regeneration of the upper arch, with simultaneous computer-guided positioning of six implants delayed. During the healing and osseointegration phase of the upper implants, a removable temporary total prosthesis was used, and the definitive lower prosthesis was finalized.

The study of the OVD and digital smile design (DSD) were the cornerstones for the design of the case. Following the discovery of stable reference points, the metal support structure was digitally designed, evaluating extensions, masticatory loads, emergence profiles, and bone height for the positioning of the fixtures. The various crucial design phases for the rehabilitation of the upper arch, more complex than the rehabilitation in the lower one, will now be analyzed in greater detail, to make the reader more aware of the completely digital workflow, and of the possibilities and potential inherent in the latest programming 3D software in dentistry.

### 2.1. Surgical Guide Design

**Figure 3:**
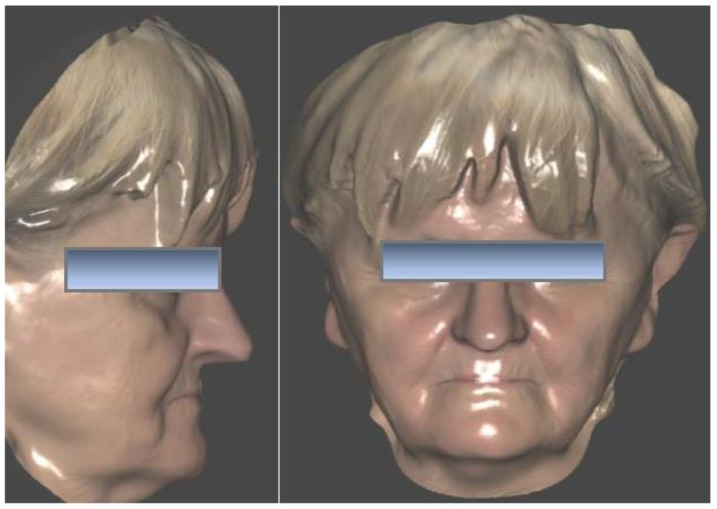
Pre-operatory facial scan.

Three months after implant surgery in the lower jaw, a low-dose CBCT was performed (with Field of View of 8 × 9 cm), using the upper removable prosthesis with three radiopaque landmarks glued on it (Medialab spa, Milan, Italy). A second CBCT of the prosthesis alone was then performed, and a STL file was obtained from the DICOM using Implant3D^®^ software (Medialab spa, Milan, Italy). Subsequently, a three-dimensional scan of the patient’s face was acquired (Figure 3) using the Bellus3D^®^ application for the IOs system (Bellus3D, Campbell, CA, USA).

**Figure 4:**
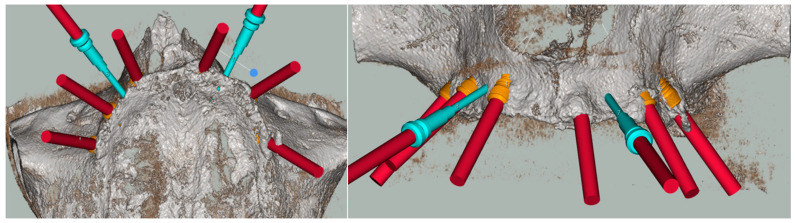
Digital Implant Planning. The numbers identify the implants and pins.

Once acquired, all the DICOM and STL files mentioned were matched and the digital surgical planning was started using the Implant 3D^®^ software (Figure 4).

The placement of seven implants (Intra-Lock System Europa, Salerno, Italy), with a diameter of 4 mm and a length ranging from 8 to 10 mm, was planned. The objective of the guided design was mainly to be able to distribute the loads over the entire arch in the most prosthetic way possible, and to obtain sufficient primary stability of the implants in the first instance.

Due to the bone scarcity of the upper arch, the clinician was concerned of not reaching primary stability in one implant site. For this reason, it was decided to add an extra implant to the surgical guide in a different area, if the minimum requirements were not met. Consequently, the implant sites guided by the template were 7, compared to the six implants needed for the prosthesis.

**Figure 5:**
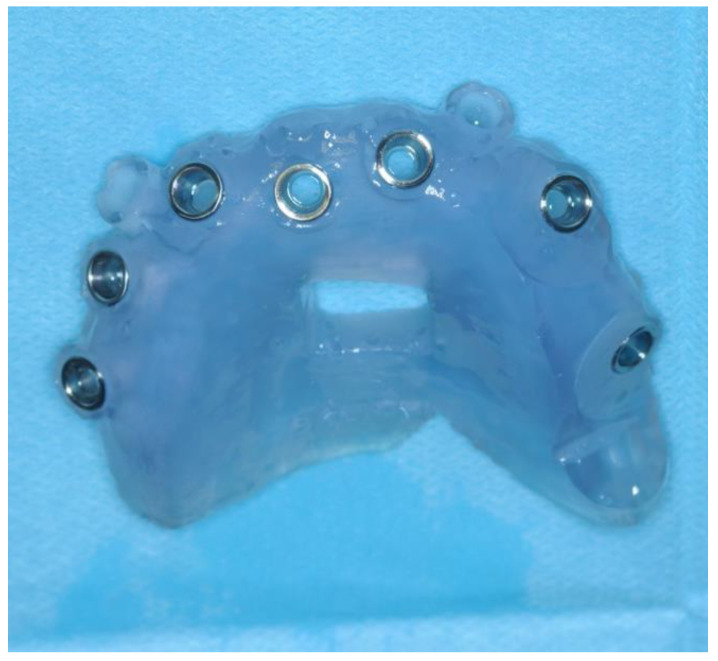
The surgical guide.

The exclusively mucosa-supported surgical template was stabilized by the design of two guides for the insertion of the stabilization pins at the level of the vestibular fornix. The surgical guide was printed using Keyprint^®^ (Keystone, Singen, Germany) on the MoonNight^®^ 3D printer (Vertysistem, Vicenza, Italy) (Figure 5).

### 2.2. Surgery

It was prescribed to take 2 g of Amoxicillin + Clavulanic Acid one hour before the operation, to be continued for five days, with 1 g every twelve hours. Four vials of Articain (1:100,000 Adrenaline) were used during the operation.

**Figure 6:**
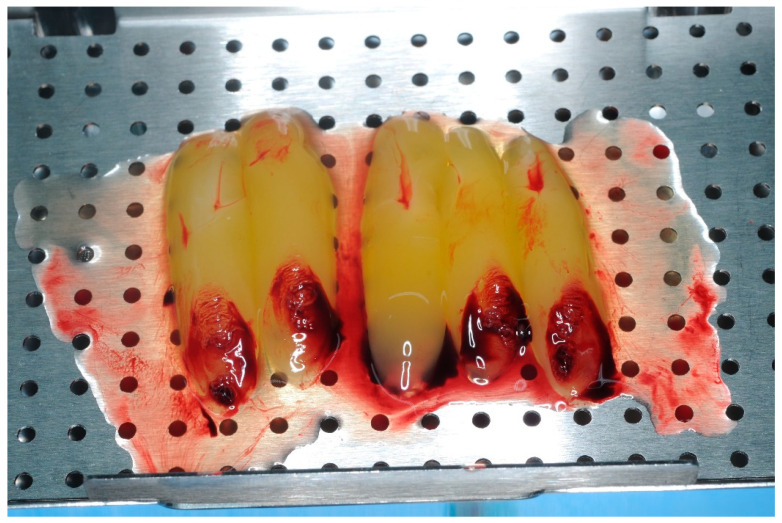
L-PRF Membranes.

Blood was drawn and eight 10 mL vials were collected and placed in the Intra-Spin^®^ centrifuge (Intra-Lock System Europa, Salerno, Italy) for twelve minutes. Following centrifugation, Leucosite Platelet-Rich Fibrin (L-PRF) membranes (Figure 6) and 1 vial of liquid PRF were obtained to be used during the bone regeneration procedure. The liquid PRF and the residual plasma derived from the compression of the PRF membranes were mixed with 1 g of bone NuOss^®^ (ACE Surgical Supply Inc., Brockton, MA, USA) to obtain the “sticky bone”.

**Figure 7:**
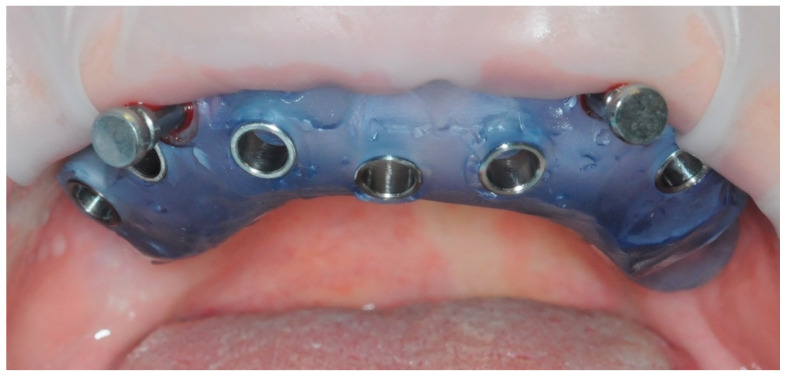
Surgical guide in place.

After one minute of rinsing with 0.20% chlorhexidine and preparation of the sterile surgical field, the fit of the sterilized and pin-stabilized surgical guide was tested (Figure 7).

**Figure 8:**
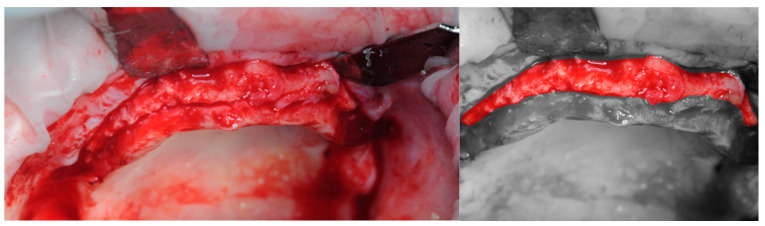
Elevation of the full thickness flap.

Once the accuracy of the template was confirmed, a full thickness flap was detached (Figure 8). The flap detachment was extended until it did not interfere with the correct positioning of the template. The template with the fixing pins was, therefore, repositioned (Figure 7).

**Figure 9:**
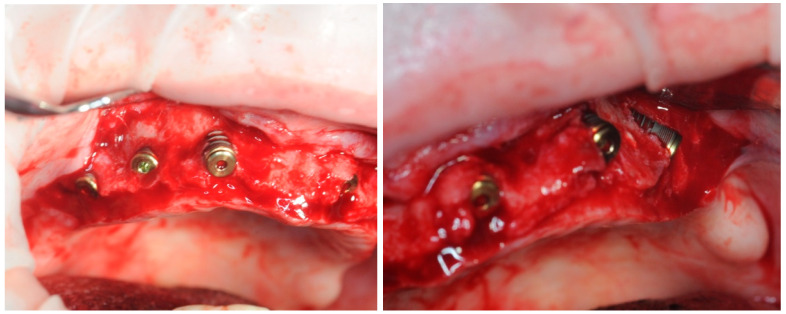
Detail of the implant positioning.

The Intra-Guide^®^ protocol (Intra-Lock System Europa, Salerno, Italy) was followed for the guided preparation of the implant sites. Considering the steep shape of the residual bone, particular care was given to the osteotomy to avoid any drift of the subsequent preparation drills. After the osteotomy, drills of increasing length (6.5 mm, 8 mm, and 10 mm, where necessary) and diameter (2.0 mm, 2.4 mm, and 2.8 mm) were used to create an adequate implant socket. All implants were screwed in with a torque of 10 or more N m (Figure 9).

**Figure 10:**
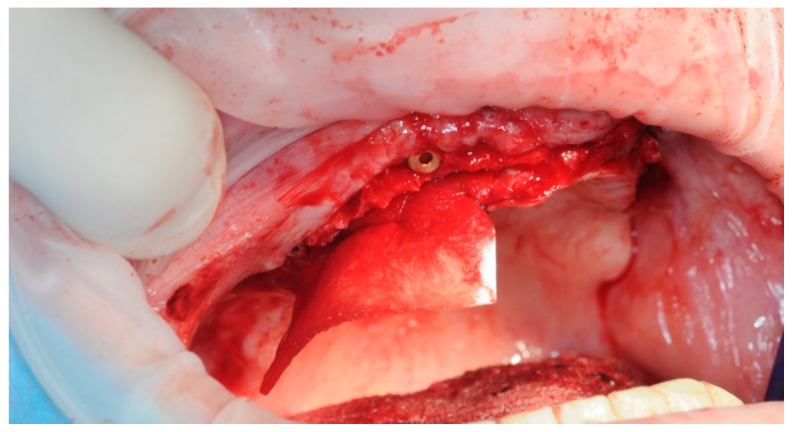
Detail of the collagen membrane.

A collagen membrane was stitched under the palatal flap, in order to provide an extra layer of protection (Figure 10).

**Figure 11:**
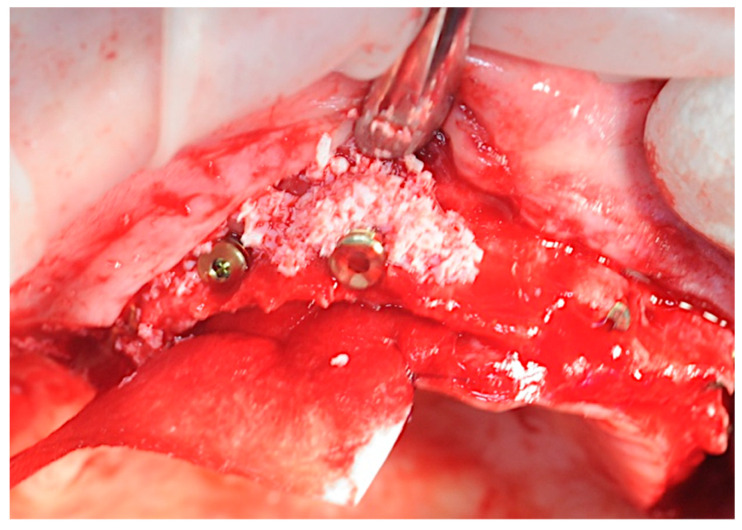
Application of the sticky bone.

The previously prepared sticky bones were placed on the exposed titanium threads and in the spaces between the implants (Figure 11).

**Figure 12:**
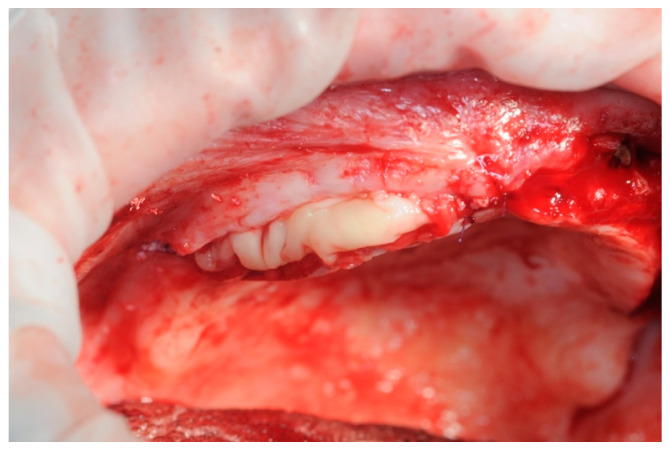
PRF membrane in place.

**Figure 13:**
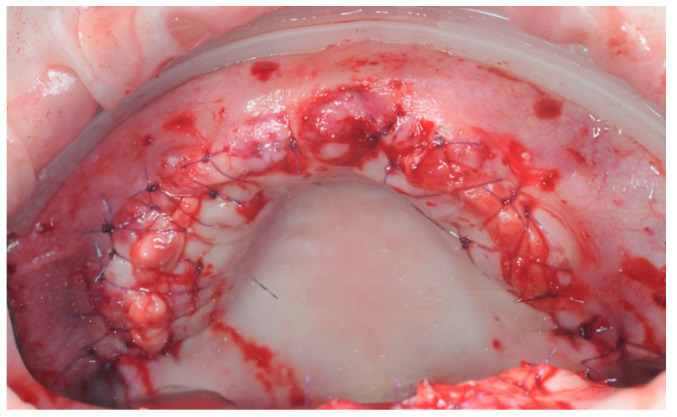
Suture of the full thickness flap with polylactic acid suture.

PRF membranes were placed (Figure 12) and the flap was closed with detached stitches 4/0 (Figure 13).

The patient reported minimal postoperative swelling, hematoma, and pain.

**Figure 14:**
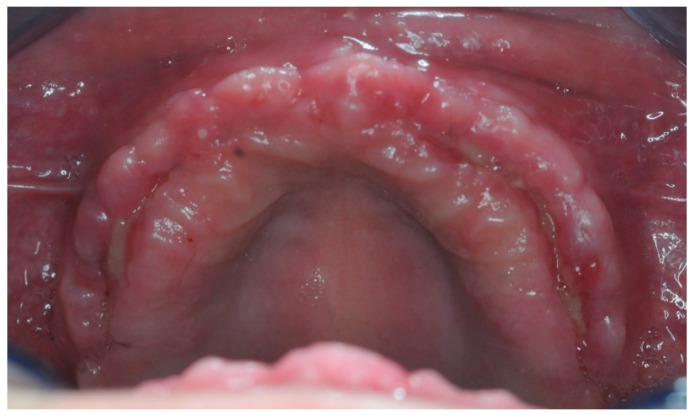
Seven days post-op.

After 7 days, the sutures were removed (Figure 14), and the patient was given a suitably modified and relined version of the removable provisional prosthesis.

### 2.3. Re-Entry Surgery

**Figure 15:**
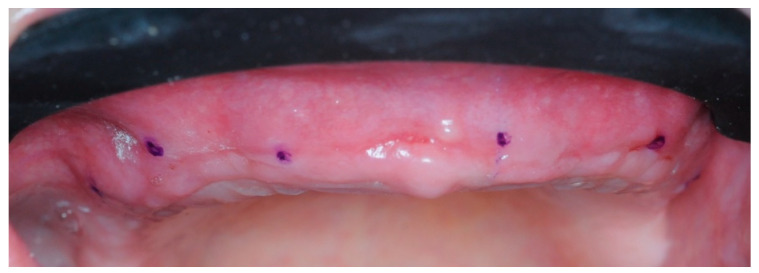
The dots indicate the approximate position of the implants.

Four months postoperatively, four 10 mL vials of blood were collected to obtain PRF membranes. Using a modified version of the same surgical guide, six points were marked on the gingiva to know the approximate position of the implants (Figure 15).

**Figure 16:**
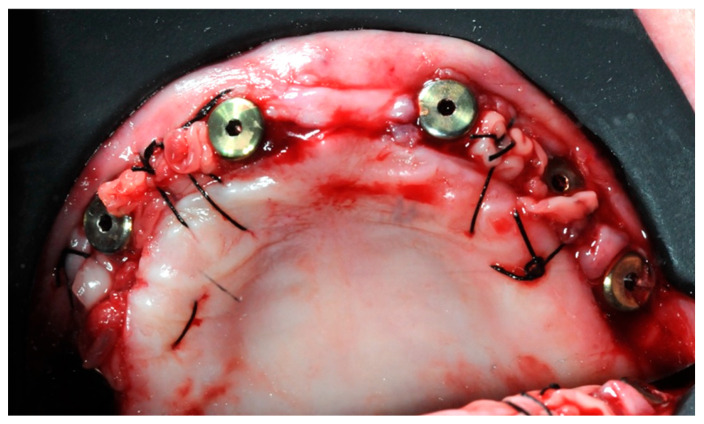
Re-entry surgery.

**Figure 17:**
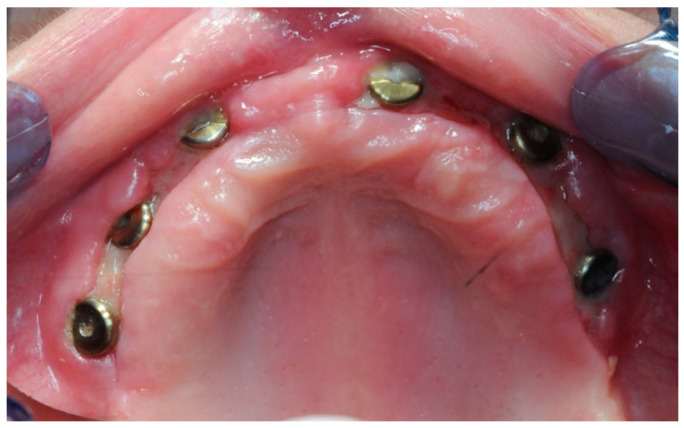
Seven days after the re-entry surgery.

After sculpting a full thickness flap, and removing the surgical screws from the fixtures, the healing abutments were screwed in. The previously prepared PRF membranes were positioned and sutured with the flap, in order to obtain secondary intention healing and increase soft tissue volumes (Figure 16). The stitches were removed after seven days (Figure 17).

**Figure 18:**
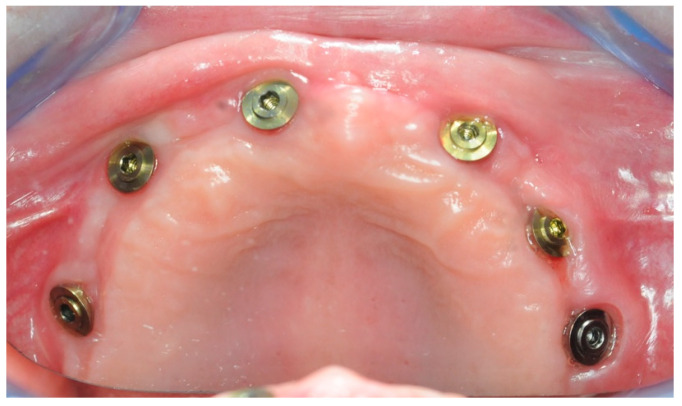
One month healing after the re-entry surgery with the Flat One^®^ abutments in place.

After one month, six 3 mm Flat One^®^ Abutments (Intra-Lock System Europa, Salerno, Italy) were screwed in (Figure 18), along with the scan abutments. Subsequently, intraoral scans were taken, in order to gather the data needed to build the temporary prosthesis.

### 2.4. Temporary Prosthesis

**Figure 19:**
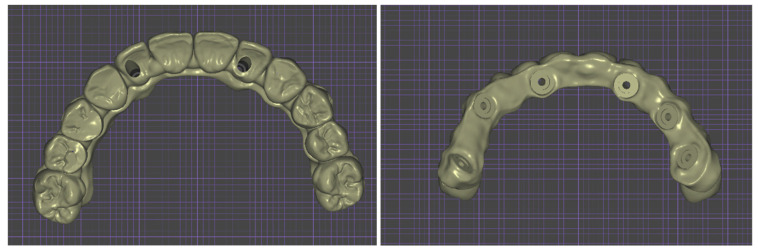
Lower and upper views of the provisional denture.

**Figure 20:**
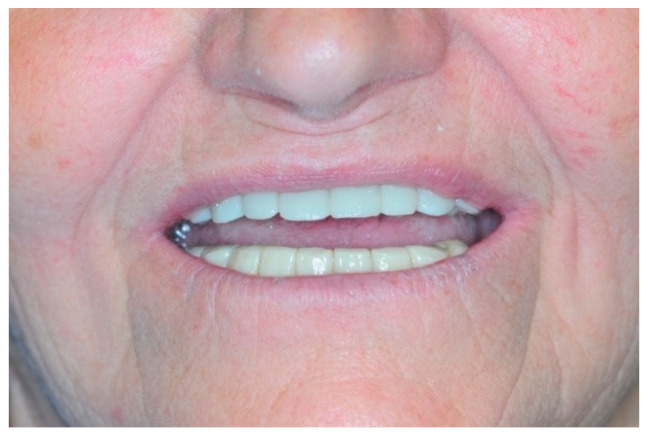
Frontal view of the provisional denture.

In order to guide soft and hard tissue healing, and for the comfort of the patient, a temporary prosthesis without abutment (Exocad^®^, Darmstadt, Germany) was created (Figure 19), 3D-printed (NextDent^®^ C&B MFH, Soesterberg, The Netherlands), and screwed directly onto the Flat One^®^ abutment (Figure 20).

### 2.5. Final Prosthesis

**Figure 21:**
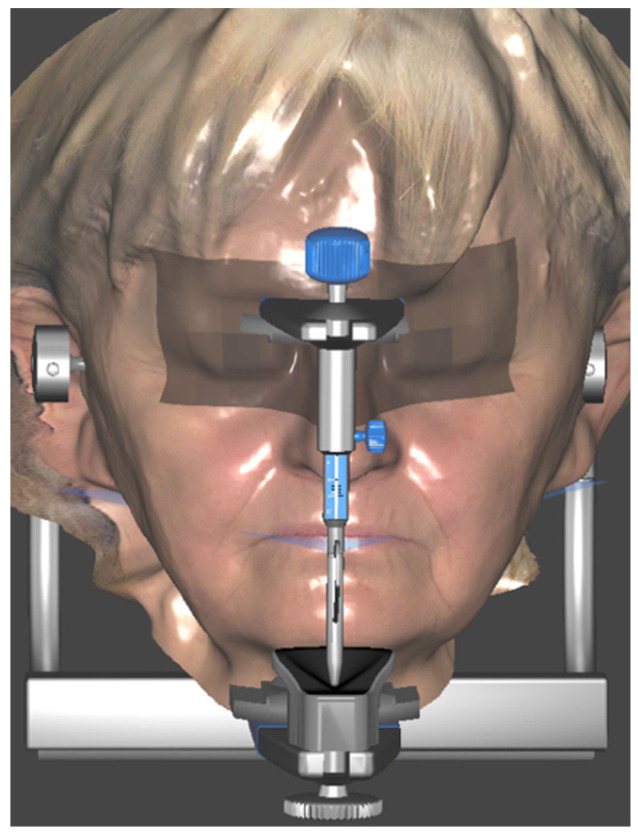
Digital Articulator Type A (Exocad^®^).

After two months, new intraoral scans of both arches, CBCT, and a new facial scan were performed. A conventional transfer facebow was used for the bite registration, and then aligned with the facial and intraoral scans in Exocad^®^ (Figure 21).

**Figure 22:**
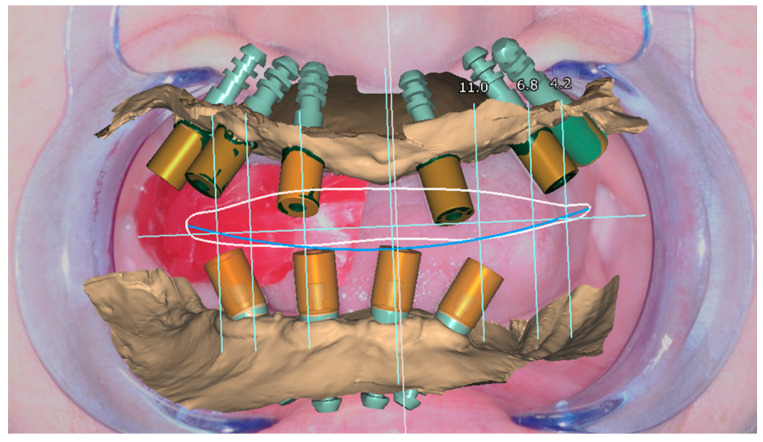
Digital Smile Design.

Thanks to the matching of photos and scans, the Digital Smile Design (DSM) was created (Figure 22).

Once the desired aesthetics had been achieved, and considering the patient’s wishes, the construction of the definitive upper and lower prosthesis began with Exocad^®^ software.

**Figure 23:**
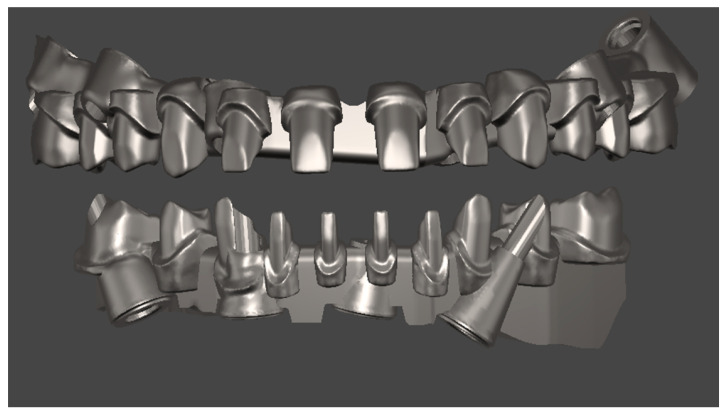
Digital design of the upper and lower substructures.

**Figure 24:**
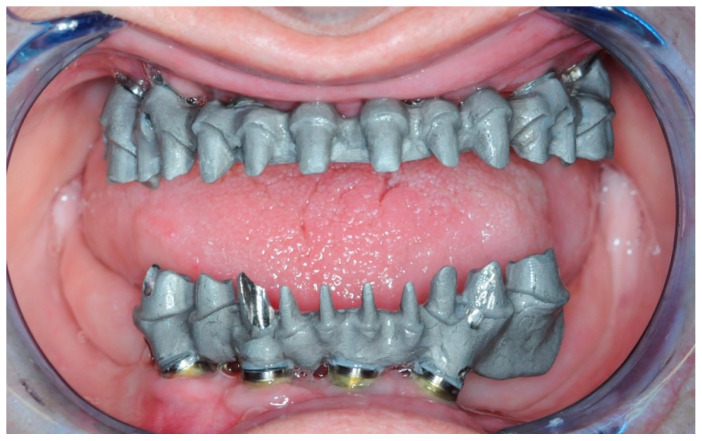
Passivization of the substructures.

The creation of the Toronto prosthesis involved the creation of the substructures in the first instance. These were then 3D-printed with Vertys Cast 3D^®^ (Vertysistem, Vicenza, Italy) and cast using the lost wax technique in cobalt-chrome alloy (Figure 23) and tested on the patient to verify their passivity (Figure 24).

**Figure 25:**
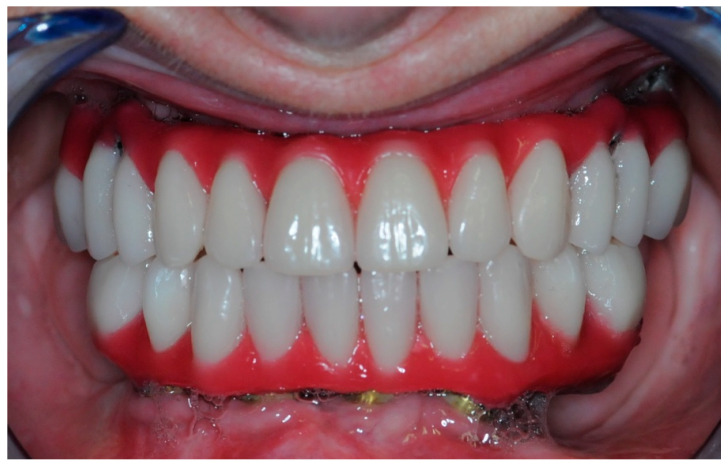
Aesthetic and Functional try-in.

The teeth were milled and glued to the metal substructure (Figure 25).

**Figure 26:**
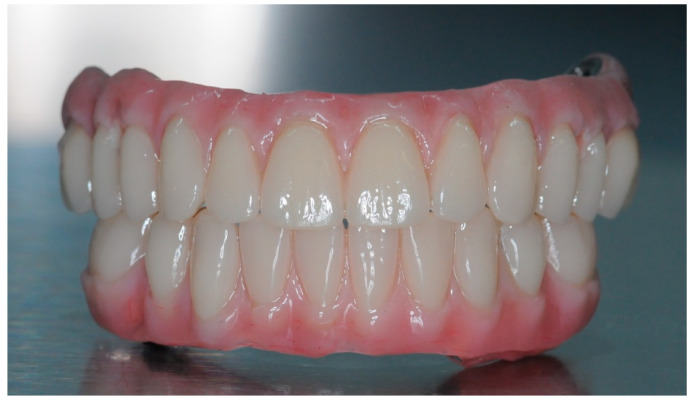
Finalized upper and lower prosthesis.

**Figure 27:**
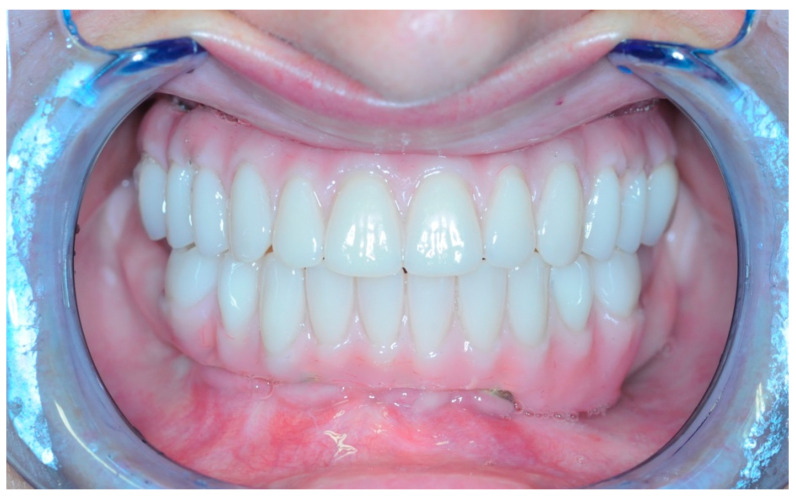
Screwed-in prosthesis.

**Figure 28:**
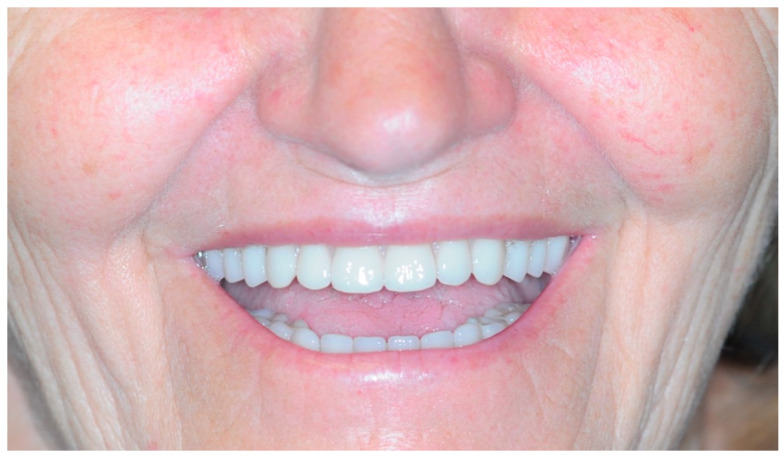
Smile.

Once the resin gingival component was added, the prosthesis was finalized for delivery (Figure 26, Figure 27 and Figure 28).

During the clinical session, the prosthesis was screwed and functionalized after requiring minimal occlusal adjustments. At this point, the end-of-case data was collected.

## 3. Results

Given the scarcity of hard and soft tissues, one crucial aspect of the rehabilitation was to find the best possible position to place the implants to achieve primary stability, and to guarantee a good emergence profile for the future prosthesis. The digital planning and subsequent accuracy of the resulting surgical guide were of pivotal importance towards these two aspects.

### 3.1. Alignment Accuracy

**Figure 29:**
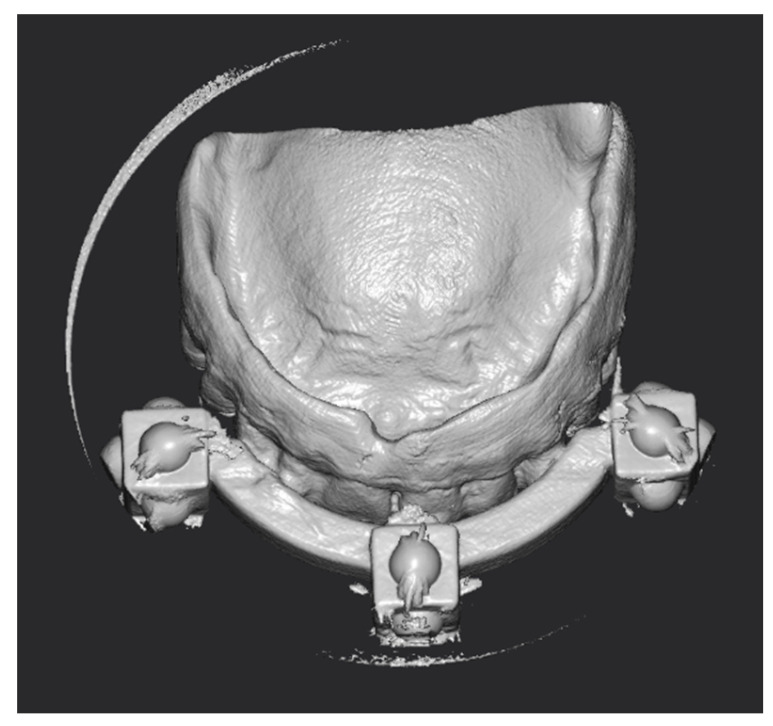
STL model obtained from the DICOM conversion.

**Figure 30:**
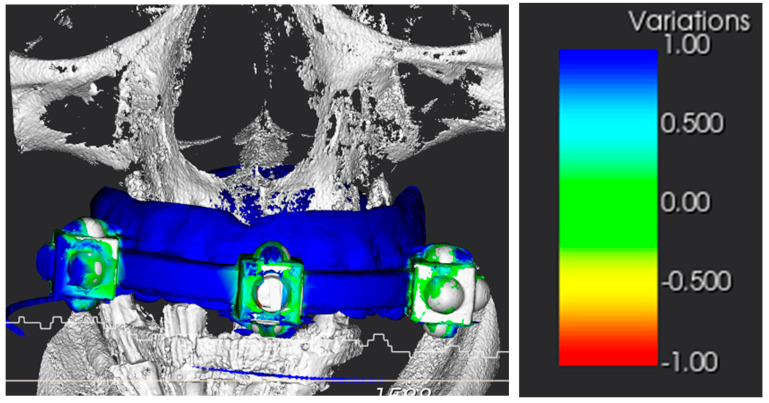
Alignment of the STL to the DICOM file and relative accuracy color scale in mm.

The conversion of the CT scan of the removable prosthesis from DICOM to STL was precise (Figure 29). Subsequently, the accuracy of the alignment between the patient’s CT scan and the landmarks in the STL file was within an acceptable margin of error (Figure 30).

### 3.2. Surgical Guide Accuracy

**Figure 31:**
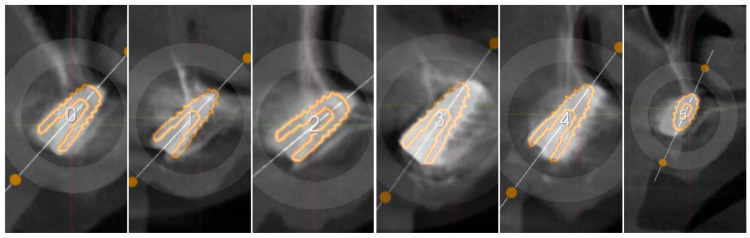
Super imposition of the digital plan and the Post-op CT scan. The numbers from 0 to 5 identify the implants placed.

All the implants achieved at least 10 N m of insertion torque, and the following discrepancies were measured for each implant compared to the digital plan (Table 1, Figure 31).

### 3.3. Hard Tissues Gain

**Figure 32:**
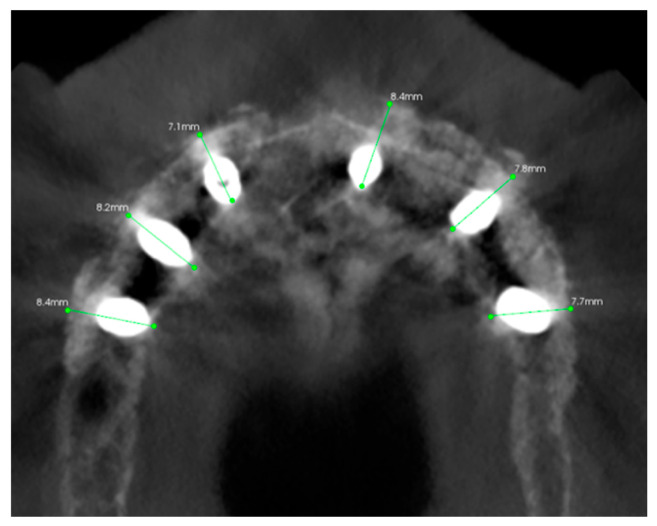
1-year control.

From the comparison between pre- and post-op and the 1-year control, it is possible to say that the amount of hard tissue gained is substantial and remained stable throughout twelve months of function (Figure 32).

### 3.4. Soft Tissue Management

**Figure 33:**
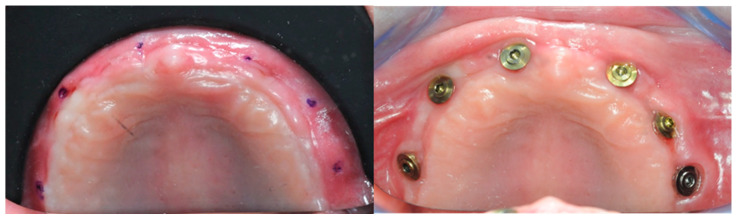
Soft tissues pre and post re-entry surgery.

In order to achieve primary stability, given the bone scarcity, compromises had to be made regarding the emergence profile. Four months after the surgery, a lack of keratinized gingival tissue was evident (Figure 33). During the re-entry surgery, the gingiva was repositioned to induce second intention healing, with the help of L-PRF membranes, and the volume of soft tissues surrounding the implants was increased (Figure 33).

### 3.5. Function and Aesthetics

**Figure 34:**
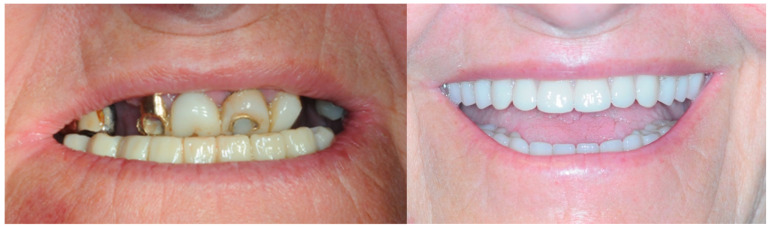
Smile comparison.

**Figure 35:**
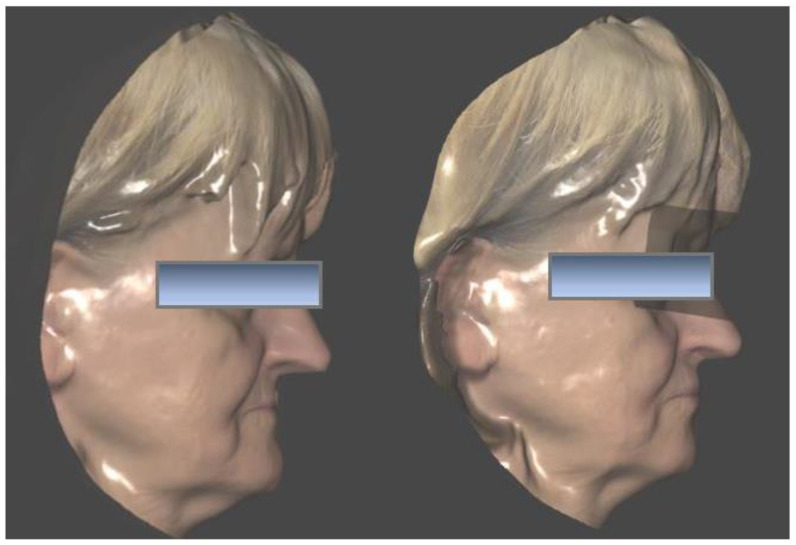
Pre- and post-op facial scan profile comparison.

After establishing the proper VOD, also taking into account the patient’s feedback during the time she spent with the provisional denture, the teeth were positioned with the help of the Digital Smile Design (Figure 22) and the substructures were finalized (Figure 23 and Figure 24). Perhaps the most stunning result, aside from the pre- and post-op smile comparison (Figure 34), is the change of the lower part of the profile, including the nose (Figure 35).

## 4. Discussion

Dental implants have radically changed the oral health outcome of patients with partial or complete edentulism [30,31,32].

Placing the implants in an optimal position is an important key to achieving a high long-term success rate, in terms of implant stability and prosthetic validity [29].

The existing literature is not rich in information; neither are protocols regarding pre-surgical digital programming, regarding prosthetic implant rehabilitation in the presence of severe atrophy of the jaws. Some studies, such as that of Maiorana et al. [1,33], have evaluated the efficacy of guided implant placement and the contextual GBR procedures. The evidence they provided demonstrates that guided implant placement is a clinically beneficial procedure, especially in patients with low bone density. Even if it has not yet been fully demonstrated, theoretically, it seems that guided positioning, by allowing the implant to be positioned in an optimal way, with respect to the present bone and the prosthetic needs, can partially reduce the amount of bone regeneration required [1]. Another positive aspect of digital programming could be demonstrated: the use of stereolithographic templates to guide the position of the implant leads to a reduction in the duration of surgical procedures [34,35,36]. Therefore, many procedures are possible with virtual planning, including: determination of the implant position, guided pre-drilling of the implant socket, pre-counting of the membranes for GBR, and preliminary production of the provisional prosthesis, in case of immediate loads. For the reasons illustrated, the times of surgical and prosthetic procedures can be significantly reduced. The literature currently shows the potential of these tools; however, there is a lack of protocols that are truly full-digital in all phases. The achievement of the virtual design requires the acquisition of a series of digital data: first of all, the digital intraoral scan of the dental arches. The effectiveness of using intraoral scanners has been demonstrated and well-documented in recent years, compared to the traditional method of taking impressions with materials such as alginate [37,38]. Digital impressions in general are accurate, more comfortable for the patient, allow for the realistic capture of soft tissue anatomy, and are less prone to pressure, expansion, and distortion-induced problems than traditional impression materials. However, the scanners currently on the market are not entirely insensitive to the manual dexterity of the operator: the use of an incorrect technique or errors in the acquisition phase of the images can generate a certain degree of distortion of the stereolithographic models. A lack of congruence between the patient’s arches and the stereolithographic model necessarily results in a deficit in the accuracy of the virtual implant plan [39,40].

To carry out the computer-guided digital project, it is essential to acquire a three-dimensional radiography of the jaw bones, nowadays often performed using the cone beam computer tomography instrument (CBCT). In recent years, the validity of this radiological tool has been extensively studied [41]. The CBCT, born mainly to satisfy the needs of the dental field, has been evaluated, above all, in terms of precision, reliability and real emission of a low radiation dose [42,43,44]. Pre-surgical digital programming relies upon the superimposition of hard and soft tissues, achieved through the aligning of the STL, PLY, OBJ or other file extensions, depending on the intraoral scanner and the DICOM file from the CT scan.

For the successful rehabilitation of an edentulous arch, especially in the presence of severe maxillary atrophy, the digital planning needs to account for not only the proper implant positioning, in regards to the available bone tissue, but also to the soft tissues’ emergence profiles, and the bio-mechanical and aesthetic needs of the prosthesis. These requirements, along with the passive fitting of the substructure and the proper VOD, are crucial, in order to guarantee the success of the rehabilitation in the long-term [45,46].

The computer-guided surgical systems available on the market have been extensively studied and compared with the traditional method. Today, most of the critical issues related to the accuracy and the cost-benefit problem can be considered overcome [47,48,49]. Numerous studies in the literature [50,51] confirm the precision and accuracy rate of these tools. Studies have been published which confirm that guided implant placement increases the implant survival rate, compared to the freehand method [52]. Other studies also believe that the digital workflow in the implant-prosthetic field can effectively and predictively improve the immediate load rehabilitation process of completely edentulous arches [23]. From an analysis of the current literature available, it is indisputable that the greatest challenge to overcome is the capacity for reliable and efficient digital recording of the maxilla-mandibular relationship [53].

The clinical case presented here was selected due to the Inherent difficulty in designing an extremely atrophic maxilla; in this case, the three-dimensional computer-guided implant design finds the maximum degree of application in terms of optimization of the project, based on anatomical evidence and prosthetic needs, consequently, making the surgical procedure easier, safer and, last but not least, predictable. It is common finding that digitally guided techniques have made associated surgical protocols more efficient, less invasive, and with more favorable prognoses. Digital restorative workflows require precise and detailed virtual planning of anatomy and occlusion, taking into account aesthetic considerations. Planning and subsequent prosthetic production using entirely digital methods, together with the introduction of new printing and milling materials, have recently led to a substantial transformation of the prosthetic workflow. This type of workflow allows for the creation of prosthetic structures with customized smile aesthetics, as the result of the combined analysis of several determining factors: this allows the clinician to respond more appropriately to the needs and requirements of the patient. From many studies, the digital technique for programming full arches has been described as demanding and complex, due to the amount of data required. The management and processing of the files in question requires not only medical skills, but also computer skills and specific knowledge of the software used. Limitations to this approach are mainly given by the difficulty of recording that is currently encountered with existing tools, for which the transfer of analog data to a digital platform is often still used.

Therefore, these workflows are technique-sensitive and complex [54,55]: to reduce the risk of significant limitations in clinical performance, and to compensate for any limitations, auxiliary scan guides, markers and pins have been developed that can represent additional anatomical markers in the scanning phase, and, ultimately, facilitate the occlusal and maxillomandibular registration.

Many studies [56] have asserted that computer-guided placement is not only faster, but also safer for implant placement. These conclusions were reached through overlapping studies of pre- and post-operative CBCT in relation to the initial implant design. These evaluations were also carried out for the case under discussion, arriving at the same conclusions.

Mucositis and peri-implantitis are medium-long term biological complications with multifactorial causes [57,58]. The lack of respect for the soft tissues determined by an incongruous implant treatment plan is one of the possible causes determining the pathology [59]. Recent digital solutions limit the onset of this type of problem, as they allow the insertion site to be planned and prepared, avoiding and preventing the greatest number of prosthetic compromises, and improving the level of precision and accuracy [60,61,62,63,64].

These technologies offer the clinician the resolution of complex clinical pictures by hypothesizing and pre-visualizing multiple treatment plans: digital programming offers more predictable results.

The evaluation of the positioning accuracy of dental implants compared to digital planning is performed with a method validated by many scientific studies. Using special software, the superimposition of the preoperative CBCT with planned implants, and the postoperative CBCT with positioned implants, is performed. The delta, i.e., the discrepancy obtained from the superimposition, represents the margin of error of the guided surgical method [65,66,67,68,69].

However, performing a CBCT scan after implant placement, in the absence of signs and symptoms, represents a biological cost justified for study reasons, so it is necessary to carefully inform patients orally and in writing. However, this is essential to understand whether the method used is sufficiently accurate and consequently safe and predictable, evaluating the existing discrepancies between planning and implementation. To date, to evaluate implant deviations, the combination of a pre-operative and post-operative CBCT is the validated, most-used and published method. It is universally recognized that, in order to obtain good therapeutic efficacy, it is not only necessary to position the implants correctly, but it is also essential to position a sufficient number of them to allow correct dissipation of the masticatory forces. The need to increase the number of implants supporting the prosthesis determines their necessary positioning in the more posterior area. This may conflict with the often-reduced amount of bone, due, in part, to hyper-pneumatization of the maxillary sinus, as well as the bone regenerative procedures’ use of synthetic bone and collagen and/or PRF membranes. Growth factors play an important role in the healing process, and in the almost completely asymptomatic postoperative course. The advantages to be attributed to the use of membranes and derivatives of the patient’s blood are considerable. Of interest is a 2017 systematic literature review by Chouchroun et al. [70] on the use of PRF in dentistry. Oral surgery procedures in which PRF can be used lead to the formation of new bone tissue. These capabilities derive from its fibrous structure, which facilitates the retention of a significant amount of cytokines and growth factors, capable of supporting the creation of a scaffold that favors cell migration. The solid fibrin matrix created by PRF plays a fundamental role and can dissolve itself more slowly than other derivatives, such as PRP.

Another advantage of PRF, demonstrated by several studies [71], is the ability to release growth factors and cytokines at the healing site for a period of more than 10 days.

The centrifugation of the patient’s blood, necessary to avoid the coagulation cascade, is the necessary and indispensable process to obtain the fibrin reticulum, which, adequately removed from the tube, is compressed to form membranes. The fibrin-created scaffold underlies the angiogenic potential and healing facilitation that results from its use. This network can recruit circulating stem cells, to regulate the in situ immune response and to stimulate epithelialization, promoting the wound-healing and closure process.

The use of PRF in sinus lift procedures requires further study and confirmation; however, current literature reviews show that PRF used as the sole graft material promotes significant bone gain.

Used in combination with other regenerative materials, it appears to decrease the overall healing time by stimulating rapid tissue-healing, increasing the rate of recruitment and differentiation of various cell lines. Because of this, the ability to reduce the post-operative bacterial load is also not negligible.

## 5. Conclusions

Computer-assisted implantology is a safe and precise method of performing dental implants. Preliminary studies have a high degree of accuracy, but further studies are needed to arrive at a fully digital clinical protocol at all stages.

## Figures and Tables

**Table 1:** Accuracy of each implant compared with digital plan, measured superimposing pre-op and post-op CT scan.

Implant Reference	Palatal-Vestibular Accuracy (From the Apex of the Implant)	Mesio-Distal Accuracy (From the Apex of the Implant)	Height Accuracy (From the Apex of the Implant)
Implant 0	0.5 mm	1 mm	0.7 mm
Implant 1	0.3 mm	0.4 mm	0.2 mm
Implant 2	0.3 mm	0.2 mm	0.4 mm
Implant 3	0.9 mm	1.1 mm	0.9 mm
Implant 4	0.7 mm	1 mm	0.4 mm
Implant 5	0.8 mm	1.2 mm	0.2 mm

## Data Availability

Not applicable.
